# Cannabinoid CB_2_ Receptors Modulate Microglia Function and Amyloid Dynamics in a Mouse Model of Alzheimer’s Disease

**DOI:** 10.3389/fphar.2022.841766

**Published:** 2022-04-27

**Authors:** Samuel Ruiz de Martín Esteban, Irene Benito-Cuesta, Itziar Terradillos, Ana M. Martínez-Relimpio, M. Andrea Arnanz, Gonzalo Ruiz-Pérez, Claudia Korn, Catarina Raposo, Roman C. Sarott, Matthias V. Westphal, Izaskun Elezgarai, Erick M. Carreira, Cecilia J. Hillard, Uwe Grether, Pedro Grandes, M. Teresa Grande, Julián Romero

**Affiliations:** ^1^ Faculty of Experimental Sciences, Universidad Francisco de Vitoria, Pozuelo de Alarcón, Spain; ^2^ Department of Neurosciences, Faculty of Medicine and Nursing, University of the Basque Country UPV/EHU, Leioa, Spain; ^3^ Achucarro Basque Center for Neuroscience, Science Park of the University of the Basque Country UPV/EHU, Leioa, Spain; ^4^ Roche Pharma Research & Early Development, Roche Innovation Center Basel, F. Hoffmann-La Roche Ltd., Basel, Switzerland; ^5^ Laboratorium Für Organische Chemie, Eidgenössische Technische Hochschule Zürich, Zürich, Switzerland; ^6^ Department of Pharmacology and Toxicology, Neuroscience Research Center, Medical College of Wisconsin, Milwaukee, WI, United States

**Keywords:** cannabinoids, CB_2_ receptor, amyloid, Alzheimer’s disease, microglia

## Abstract

The distribution and roles of the cannabinoid CB_2_ receptor in the CNS are still a matter of debate. Recent data suggest that, in addition to its presence in microglial cells, the CB_2_ receptor may be also expressed at low levels, yet biologically relevant, in other cell types such as neurons. It is accepted that the expression of CB_2_ receptors in the CNS is low under physiological conditions and is significantly elevated in chronic neuroinflammatory states associated with neurodegenerative diseases such as Alzheimer’s disease. By using a novel mouse model (CB_2_^EGFP/f/f^), we studied the distribution of cannabinoid CB_2_ receptors in the 5xFAD mouse model of Alzheimer’s disease (by generating 5xFAD/CB_2_^EGFP/f/f^ mice) and explored the roles of CB_2_ receptors in microglial function. We used a novel selective and brain penetrant CB_2_ receptor agonist (RO6866945) as well as mice lacking the CB_2_ receptor (5xFAD/CB_2_^−/−^) for these studies. We found that CB_2_ receptors are expressed in dystrophic neurite-associated microglia and that their modulation modifies the number and activity of microglial cells as well as the metabolism of the insoluble form of the amyloid peptide. These results support microglial CB_2_ receptors as potential targets for the development of amyloid-modulating therapies.

## Introduction

Cannabinoid receptors include two types of G-protein coupled receptors (GPCRs), CB_1_ and CB_2_, that exhibit profound differences in their distribution in the organism of mammals (Pertwee et al., 2010). While the CB_1_ receptor is one of the most abundant GPCRs in the brain and its expression is constitutive in a wide variety of cells and tissues, the distribution of CB_2_ receptors is restricted to specific types of cells (B-lymphocytes, natural killer cells, monocytes, etc) and tissues (spleen, Peyer’s patches) and its brain expression is low under physiological conditions (for review, see Mechoulam and Parker, 2013). Importantly, the expression of cannabinoid CB_2_ receptors is significantly increased under pathological conditions and, specifically, in the context of chronic neuroinflammation (Maresz et al., 2005; Mecha et al., 2016).

The pattern of expression of CB_2_ receptors and its biological relevance in the CNS is still a matter of debate. It is currently accepted that microglial cells express CB_2_ receptors under both normal and pathological conditions (Komorowska-Müller and Schmöle, 2020). Interestingly, although its presence in neuronal elements is believed to be low (if any), there are reports that CB_2_ receptors contribute to functions ascribed to neurons, such as pain or reward (Zhang et al., 2014; Cabañero et al., 2020; He et al., 2021). CB_2_ receptors also seem to play important roles in neurodegenerative conditions, although their precise contribution has not been elucidated yet due to conflicting results (Mecha et al., 2016; Galán-Ganga et al., 2021; Rodríguez-Cueto et al., 2021).

The amyloid hypothesis of Alzheimer’s disease (AD) is currently the most widely accepted among the scientific community (Hampel et al., 2021). Together with hyperphosphorylated tau-enriched neurofibrillary tangles, neuritic plaques (primarily constituted by amyloid peptides and, specifically, beta amyloid 1-42, Aβ) are the main pathologic features of AD. Multiple deleterious consequences derive from the accumulation of both in the brain, including mitochondrial dysfunction, axonal degeneration, alterations in synaptic transmission and neuroinflammation (Hampel et al., 2021).

There is an urgent need for novel approaches for the treatment of this devastating disease. Efforts have been focused on tackling the neuroinflammatory process triggered by the presence of pathological forms of Aβ as it is presently thought that these peptides possess intrinsic pro-inflammatory properties that play a crucial role in the loss of neurons in specific areas of the AD brain. This process involves several types of cells (microglia, astrocytes) and mediators (cytokines, reactive oxygen species, lipids) that, acting in a concerted and time-dependent manner, expand the damage initiated by neuritic plaques and neurofibrillary tangles (see Hampel et al., 2021, for review).

Microglia seem to play a prominent role in this scenario. In the healthy brain, these cells of myeloid origin are continuously sensing their surrounding environment (Nimmerjahn et al., 2005). When an alteration takes place in the brain parenchyma, these cells become “activated”, and shape their phenotype to cope with this alteration by modifying their structural properties, gene expression profile, ability to produce cytokines and other cell mediators, and phagocytic activity (becoming “damage-associated microglia”, DAM; Deczkowska et al., 2018). Among other adaptations, microglia express cannabinoid CB_2_ receptors in the context of AD (Mecha et al., 2016), and remarkably, in neuritic plaque-associated microglia (Benito et al., 2003).

In the present study, we analyzed the expression of cannabinoid CB_2_ receptors in cortical areas of the brain of an AD mouse model (5xFAD/CB_2_^EGFP/f/f^) by electron microscopy. In addition, we explored the potential roles of this receptor through its activation with a selective agonist (RO6866945) and through its genetic deletion (5xFAD/CB_2_^−/−^).

## Materials and Methods

### Mice and Treatment

Mice used in these experiments were described in our previous study (López et al., 2018) and were housed and bred in the animal facilities of Universidad Francisco de Vitoria (Pozuelo de Alarcón, Madrid, Spain). Experimental protocols met the European and Spanish regulations for protection of experimental animals (86/609/EEC and RD 1201/2005 and 53/2013) and were approved by the committee of Ethics for Animal Welfare of the Universidad Francisco de Vitoria and University of the Basque Country (M20/2015/093). Efforts were made to minimize the number and suffering of animals.

Mice co-expressing five familial Alzheimer’s disease mutations (5xFAD) were purchased from Jackson Laboratories (Bar Harbor, ME, United States; Oakley et al., 2006) on the C57BL/6J background and were mated with CB_2_^EGFP/f/f^ and CB_2_^−/−^ mice and backcrossed for at least ten generations to generate 5xFAD/CB_2_^EGFP/f/f^ and 5xFAD/CB_2_^−/−^ mice.

Prior to the experiment, mice were homogenously distributed per group according to bodyweight. A stock solution of 90 mg/ml RO6866945 (Roche Pharma Research and Early Development, Roche Innovation Center Basel, Basel, Switzerland) in ethanol was conserved at −20°C, and diluted in vehicle solution [5% ethanol, 5% kolliphor (Sigma, C5135), 90% NaCl 0.9% (Braun, 857367)] the day of use. 6 months old 5xFAD/CB_2_^EGFP/f/f^ and 5xFAD/CB_2_^−/−^ male mice were treated (i.p.) with RO6866945 10 mg/kg, or vehicle (VEH) daily for 28 days. RO6866945 ((3*S*)-1-[5-*tert*-butyl-3-[(4-methyl-1,2,5-oxadiazol-3-yl)methyl]triazolo[4,5-d]pyrimidin-7-yl]pyrrolidin-3-ol; CAS Registry Number 1433360-72-5) was synthesized as described in US20130116236 A1 (Example 136) (Adam et al. (2013). Preparation of [1,2,3]triazolo[4,5-d]pyrimidine derivatives useful as cannabinoid receptor 2 agonists, *US20130116236 A1*). It is a highly potent CB_2_ agonist across species (human CB_2_ cAMP EC_50_ 0.2 nM, 104% efficacy; mouse CB_2_ cAMP EC_50_ 0.2 nM, 101% efficacy) which does neither interact with the CB_1_ receptor in the cAMP (human CB_1_ cAMP EC_50_ > 10′000 nM) nor in the radioligand binding assay (human CB_1_ Ki > 10′000 nM; Ouali Alami et al., 2018). RO6866945 exhibits an excellent early ADME profile including an oral bioavailability of 44% in mice and penetrates through the blood brain barrier.

Twenty-four hours before the end of the treatment, mice were intraperitoneally injected with 10 mg/kg methoxy-X04 (Tocris, 4920) in 15% DMSO, 15% kolliphor and 70% NaCl 0.9%. Then, mice were anaesthetised with 170 mg/kg ketamine (Richter Pharma, 580393.7) and 10.7 mg/kg xylazine (Calier, 572599.4) in NaCl 0.9%, and transcardially perfused with cold PBS pH 7.4. From each mouse, right cortex, hippocampi and cerebellum were dissected and stored at −80°C. Left cortex and the rest of the brain were immediately processed to isolate microglia for analysis by flow cytometry.

### Preservation of Brain Tissue for Immunocytochemistry

Three male CB_2_^EGFP/f/f^ and three 5xFAD/CB_2_^EGFP/f/f^ mice were anaesthetized with ketamine/xylazine (100mg/10 mg/kg body weight, intraperitoneal injection) and subsequently perfused transcardially at room temperature (RT) with 4% formaldehyde (freshly depolymerized from paraformaldehyde), 0.2% picric acid and 0.1% glutaraldehyde in PBS 0.1 M (pH 7.4) for 10–15 min. The brains were then removed from the skull, post-fixed in the fixative solution for 1 week at 4°C and cut into 50 μm thick coronal sections using a vibratome.

### Double Pre-Embedding Immunogold and Immunoperoxidase Method for Electron Microscopy

Our protocol previously published was used (Puente et al., 2019). Brain sections containing the subiculum were pre-incubated in a blocking solution of 10% bovine serum albumin (BSA), 0.02% saponin and 0.1% sodium azide in Tris-hydrogen chloride buffered saline (TBS 1X), for 30 min on a shaker at RT. Tissue was then incubated for 2 days at 4°C with both a rat monoclonal anti-GFP antibody (1:500, GF090R, Nacalai) and a rabbit polyclonal anti-Iba1 antibody (1:500, 019-19741, FUJIFILM Wako Pure Chemical Corporation) prepared in 10% BSA, 0.1% sodium azide and 0.004% saponin. After washes in 1% BSA/TBS, sections were incubated with 1.4 nm gold-conjugated goat anti-rat IgG antibody (Fab’ fragment, 1:100; Nanoprobes Inc., Yaphank, NY, United States) and with biotinylated anti-rabbit IgG antibody (1:200; Biotin-SP-AffiniPure donkey anti-rabbit IgG) diluted in 1% BSA/TBS with 0.004% saponin on a shaker for 4 h at RT. They were washed in 1% BSA/TBS and then incubated with the avidin-biotin peroxidase complex (1:50; Elite, Vector Laboratories, Burlingame, CA, United States) for 1.5 h at RT. Sections were then washed in 1% BSA/TBS and kept in the same washing solution overnight at 4°C, postfixed with 1% glutaraldehyde in TBS for 12 min at RT and washed in double distilled water. Gold particles were silver-intensified with the HQ Silver kit (Nanoprobes Inc., Yaphank, NY, United States) in the dark for 12 min at RT. The biotinylated antibody was exposed to 0.05% diaminobenzidine (pH 7.4) with 0.01% hydrogen peroxide for 3.5 min at RT. Sections were incubated with 1% osmium tetroxide, pH 7.4, in the dark for 20 min, washed in PB 0.1 M, dehydrated and embedded in Epon 812 resin. 50 nm-thick sections were cut with an ultra-diamond knife (Diatome United States) and collected on nickel mesh grids. They were counterstained with 2.5% lead citrate for 20 min and examined with a transmission electron microscope (JEOL JEM 1400 Plus, Canada). Tissue was photographed using a Hamamatsu FLASH digital camera inserted in the electron microscope. Anatomical landmarks were taken to locate the subiculum region.

To ensure homogeneous labelling between all samples, only the first 1.5 µm from the section surface of each specimen was collected. Random electron micrographs were taken of the subicula. Areas of 3,524 μm^2^ in CB_2_^EGFP/f/f^ and 4,078 μm^2^ in 5xFAD/CB_2_^EGFP/f/f^ mice were examined to assess CB_2_ receptors in Iba1-positive microglia. GFP gold particles were counted and differentiated between their localization in membrane (between 0 and 30 nm of the membrane) or cytosol (more than 30 nm). Minor contrast and brightness adjustments were made to the figures using ImageJ software (NIH; RRID: SCR_003070), Adobe Photoshop and Gimp.

### Isolation of Microglial Cells and Flow Cytometry

Flow cytometry was employed to determine the ability of microglial cells to phagocytize Aβ (stained with methoxy-X04), and the levels of CB_2_ with RO7246360 probe (compound 3b in Sarott et al., 2020). 6-month-old animals were injected i. p. with Methoxy-X04 (Tocris Bioscience) at 10 mg/kg body weight. 24 h after injection, animals were deeply anesthetized by i. p. administration of a mixture of ketamine (170 mg/kg) and xylazine (10.7 mg/kg) and transcardially perfused with cold PBS 1X, pH 7.4. Brains were dissected and enzymatically digested to facilitate microglia separation. The cell suspension was mechanically dissociated and filtered through a 70 µm-cell strainer. Microglial cells, isolated by percoll gradient (GE Healthcare), were washed with PBS 1X and blocked with 1% BSA/PBS 1X for 20 min. Cells were stained with CD11b-PE and CD45-APC antibodies and with RO7246360 fluorescent probe for 40 min. Samples were read on a MACSQuant Flow Cytometer and analysed with MACS Quantify software (Miltenyi Biotec).

Debris and aggregates were eliminated from analysis by forward and side scatter characteristics. Then microglia were identified as CD11b^+^ CD45^lo^. The CB_2_ receptor expression was determined by the fluorescent signal of RO7246360 probe. Fluorescence signals were corrected by fluorescence minus one (FMO) control. For each hemisphere, approximately ten thousand CD11b + singlets were analysed.

### Cyclic Adenosine Monophosphate Assay

Extracts from frozen brain cortices were obtained by homogenization in magnesium lysis buffer (MLB: 25 mM HEPES, pH 7.5, 150 mM NaCl, 1% Igepal CA-630, 10 mM MgCl_2_, 1 mM EDTA) containing 10% glycerol, and protease and phosphatase inhibitors (1 mM Na_3_VO_4,_ 25 mM NaF and protease inhibitor cocktail; Roche) and were maintained at 4°C. Homogenates were centrifuged at 12000 g for 20 min at 4°C and supernatants were collected to determine their protein content by BCA protein assay (Pierce™ BCA protein assay kit, Thermo Scientific). Homogenates were used to measure cAMP levels using an ELISA kit (cat.no. ab65355, Abcam) following the manufacturer’s instructions. Standards and samples were plated in duplicate, and the absorbance was measured at 450 nm using a Varioskan Flash multifunction plate reader (Sunrise, Tecan).

### Aβ_1-42_ Peptide Quantification

Frozen mouse brain cortices were homogenized in four volumes (weight: volume) of TBS extracting buffer (140 mM NaCl, 3 mM KCl, 25 mM Tris, pH 7.4, 5 mM EDTA and protease inhibitor cocktail; Roche). Homogenates were centrifuged at 16,000 g for 20 min at 4°C. The supernatants were saved to quantify the soluble Aβ_1-42_ peptide fraction and the pellets were again homogenized in four volumes (weight: volume) of 5M guanidine 50 mM Tris-HCl pH 8. The supernatants obtained after the centrifugation step were collected to quantify the insoluble Aβ_1-42_ peptide fraction. An equal volume of PBS containing 1 mM serine protease inhibitor AEBSF (Sigma) was added to all samples and their protein content was determined by micro-BCA protein assay (Micro BCA™ protein assay kit, Thermo Scientific). Human Aβ_1-42_ Ultrasensitive ELISA kit (cat.no. KHB3544 Invitrogen) was used for the quantification of soluble and insoluble fractions of Aβ_1-42_ peptide following the instructions provided by the manufacturer. Standards and samples were plated in duplicate, and the absorbance was measured at 450 nm using a Varioskan Flash multifunction plate reader (Sunrise, Tecan).

### Western Blotting

Extracts from frozen brain cortices were obtained following the procedure previously described for cAMP assay. Lysates (60 μg/lane) were separated by SDS-PAGE and transferred onto nitrocellulose membranes (BioRad) and PVDF membranes (BioRad, used for the transference of phosphorylated proteins). After blocking in 5% bovine serum albumin in TTBS (10 mM Tris pH 7.5, 150 mM NaCl, 0.1% Tween 20) membranes were incubated overnight at 4°C, as appropriate, with primary antibodies: anti-phospho-p38 MAPK (1:1000; Cell Signaling Technology, 4511T), anti-p38 MAPK (1:1000; Cell Signaling Technology, 8690T), anti-phospho-CREB (1:1000; Cell Signaling Technology, 9198S), anti-CREB (1:1000; Cell Signaling Technology, 9197T), anti-phospho-ERK1/2 MAPK (1:1000; Cell Signaling Technology, 9101S), anti-Erk1/2 MAPK (1:1000; Santa Cruz Biotechnology, sc514302), Anti-Iba1 (1:1000, FUJIFILM Wako Pure Chemical, 016-20001), Anti-APP N-terminus (1:1000, EMD Millipore, MAB348), Anti-APP C-terminus (1:2000, Sigma, A8717), anti-BACE1 (1:500, Abcam, ab 2077) and anti-GAPDH (1:1000; Abcam, ab8245). Membranes were incubated with corresponding horseradish peroxidase (HRP)-conjugated secondary antibody anti-mouse IgG-HRP (1:10000; Abcam, ab97046), anti-rabbit IgG-HRP (1:5000; Cell Signaling Technology, 7074S) and were developed using a chemoluminiscent reagent (Western Lighting ECL Plus, PerkinElmer, NEL103001EA) in the appropriate equipment (ChemiDoc, Bio-Rad). GAPDH was used as an internal control. The relative quantity of protein levels in western blot was measured using ImageJ software (ImageJ; NIH).

### Statistical Analyses

All statistical analyses were performed, and graphs were generated using GraphPad Prism v 9.0 (GraphPad). Graphs represent average values ± standard error of the mean. Normality of data distribution was determined with the Shapiro-Wilk or the D’Agostino-Pearson tests. For GFP labeling, data were analyzed by means of the Mann-Whitney U test. For the rest of determinations, data were analysed by means of two-way ANOVA, followed by Tukey’s post-hoc tests. A *p*-value < 0.05 was considered as statistically significant. Only male animals were used in the experiments. The number of animals used for each experiment is reported in the figure legends.

## Results

### Microglial Localization of the CB_2_ Receptor in the Subiculum of CB_2_^EGFP/f/f^ and 5xFAD/CB_2_^EGFP/f/f^ by Electron Microscopy

**FIGURE 1:**
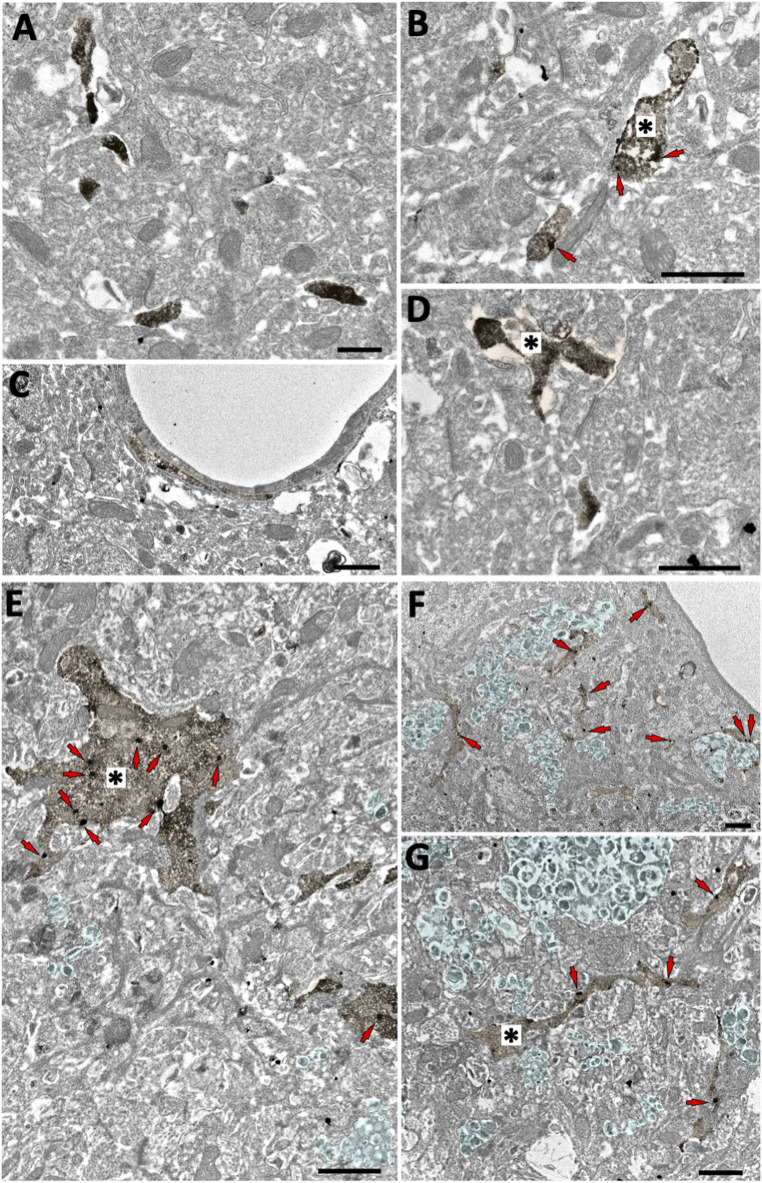
Microglial GFP localization in the subiculum of CB_2_^EGFP/f/f^ and 5xFAD/CB_2_^EGFP/f/f^ mice. Double pre-embedding immunogold (GFP) and immunoperoxidase (Iba1) method for electron microscopy. GFP particles (red arrows) localize in Iba1-positive microglial elements (DAB immunodeposits, brown, *). In CB_2_^EGFP/f/f^ **(A–D)**, only GFP membrane localization is observed (arrows, **(B)**. In 5xFAD/CB_2_^EGFP/f/f^, GFP particles are found in both membranes and cytosol **(E–G)**. Notice dystrophic neurites (light green areas contoured by white dashed lines) in 5xFAD/CB_2_^EGFP/f/f^. Scale bars: 1 µm.

**FIGURE 2:**
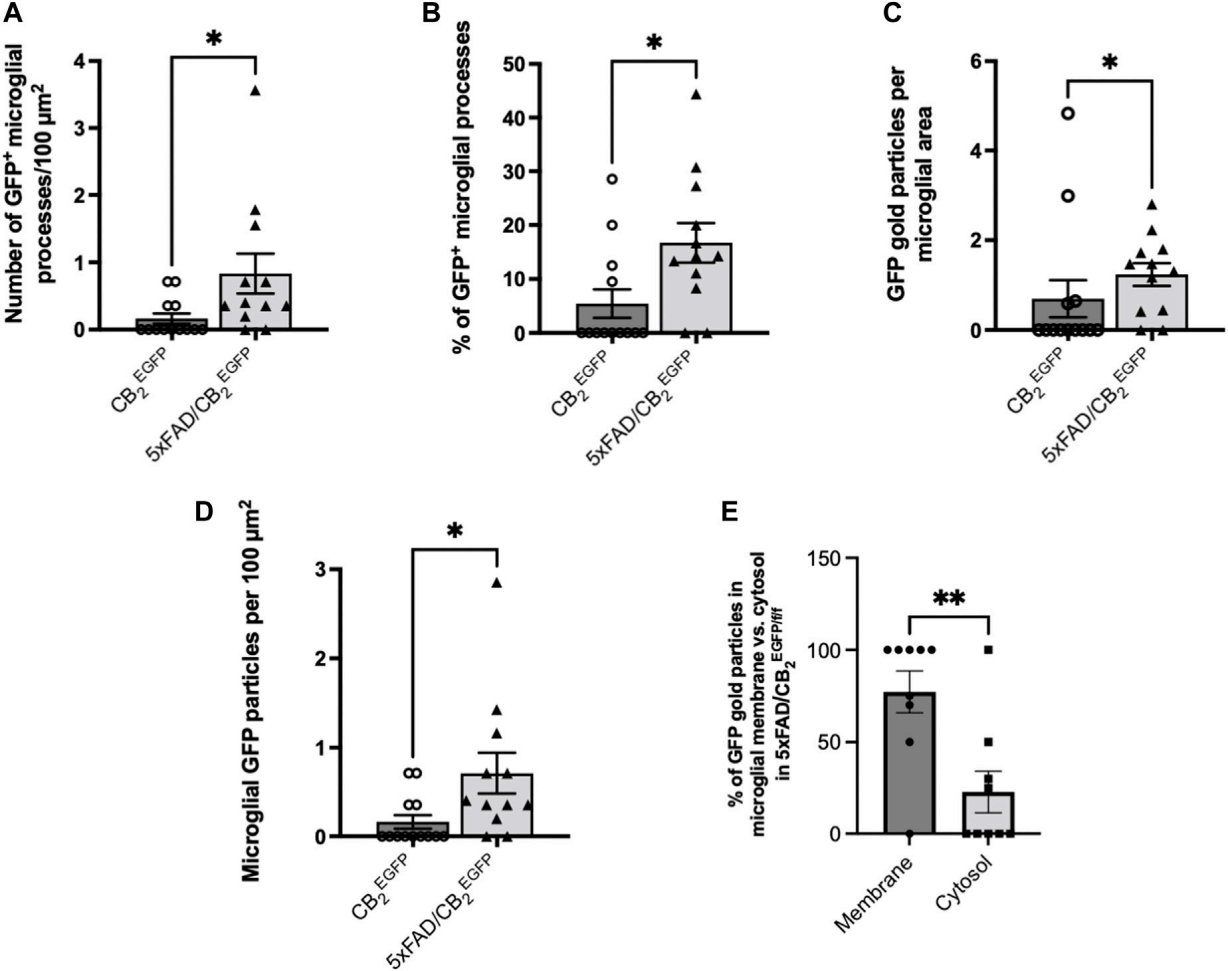
Assessment of the microglial GFP/CB_2_ localization in the subiculum of CB_2_^EGFP/f/f^ and 5xFAD/CB_2_^EGFP/f/f^ mice. **(A)** Number of microglial GFP-positive processes per 100 μm^2^. **(B)** Percentage of GFP-positive microglial processes. **(C)** GFP gold particles per microglial area. **(D)** Microglial GFP particles per 100 μm^2^. **(E)** Percentage of GFP particles in microglial membrane vs. cytosol in 5xFAD/CB_2_^EGFP/f/f^ mice. Mann-Whitney U test. *<*p*0.05; **<*p*0.01; ***<*p*0.001; ****<*p*0.0001. N = 3 mice per group. Data represent mean ± SEM.

The GFP/CB_2_ labelling was localized in Iba-1 immunopositive microglial processes in both CB_2_^EGFP/f/f^ and 5xFAD/CB_2_^EGFP/f/f^ mice (Figure 1). GFP-positive microglial processes increased significantly in 5xFAD/CB_2_^EGFP/f/f^ (0.7126 ± 0.2311) relative to CB_2_^EGFP/f/f^ (0.1648 ± 0.07686, *p: 0.0176; Figure 2). Likewise, a significant increase in the proportion of GFP-positive microglial ramifications was seen in 5xFAD/CB_2_^EGFP/f/f^ (16.71 ± 3.664%) with respect to CB_2_^EGFP/f/f^ (5.430 ± 2.631%; *p* = 0.0191; Figure 2). Also, the total number of GFP particles per area of microglial ramifications was significantly greater in 5xFAD/CB_2_^EGFP/f/f^ (1.238 ± 0.2534) than in CB_2_^EGFP/f/f^ mice (0.6962 ± 0.4138; *p* = 0.0467; Figure 2), and the number of GFP particles in microglial branches per 100 μm^2^ was statistically higher in 5xFAD/CB_2_^EGFP/f/f^ (0.8343 ± 0.2962) than in CB_2_^EGFP/f/f^ (0.1648 ± 0.07686; *p* = 0.0176; Figure 2). Noticeably in 5xFAD/CB_2_^EGFP/f/f^, the percentage of GFP immunoparticles localized in microglial membranes (77.22 ± 11.40%) was significantly higher than the proportion distributed in the cytosol (22.78 ± 11.40%; *p* = 0.0106; Figure 2). As to CB_2_^EGFP/f/f^, 100% of the GFP particles were found in microglial membranes.

### RO6866945 is a Selective CB_2_ Agonist *in vivo*

**FIGURE 3:**
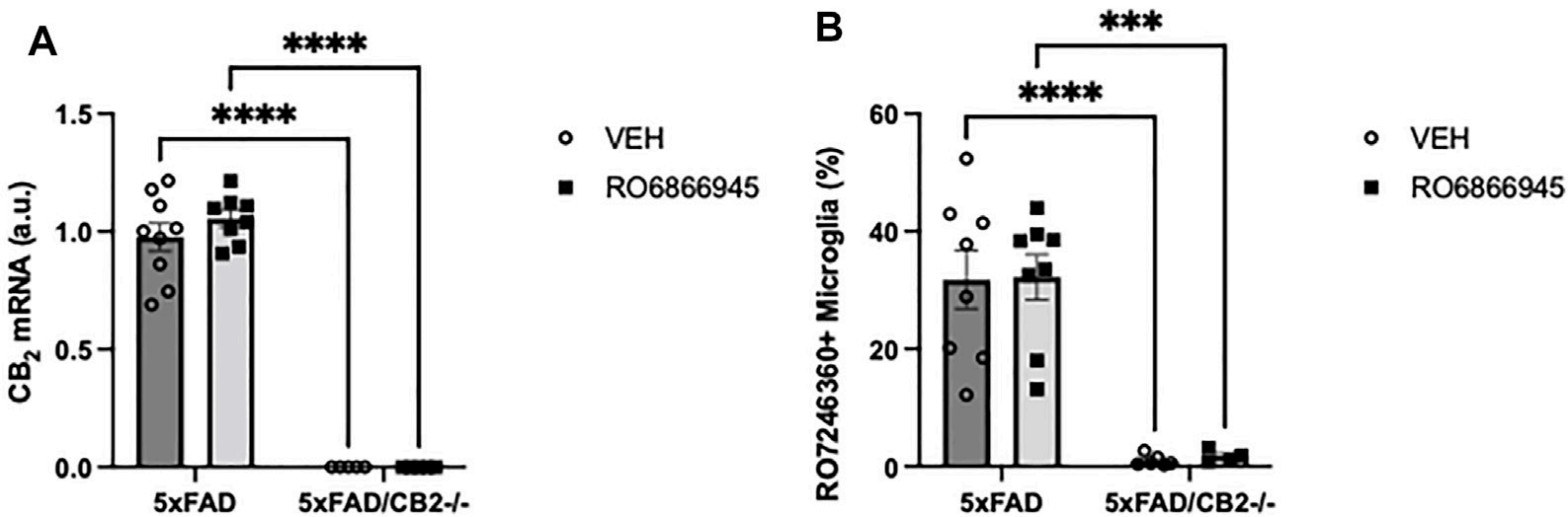
The chronic exposure to the CB_2_ selective agonist, RO6866945, did not modify the expression of cannabinoid CB_2_ receptors. **(A)** mRNA levels of the cannabinoid CB_2_ receptor did not vary after treatment with RO6866945 but were completely absent in 5xFAD/CB_2_^-/-^ mice. **(B)** Binding of the fluorescent probe RO7246360 to cannabinoid CB_2_ receptors was used to quantify protein levels, revealing no changes after treatment with the agonist and the negligible levels of CB_2_ protein in 5xFAD/CB_2_^-/-^ mice. Two-way ANOVA followed by Tukey’s post-hoc test. **<*p*0.01; ****<*p*0.0001. N = 4–9 mice per group. Data represent mean ± SEM.

We then studied whether the chronic treatment with RO6866945 had an impact on the expression levels of cannabinoid CB_2_ receptors. We used two different approaches: first, RT-PCR revealed no significant effects of the 28-days treatment with the agonist on CB_2_ mRNA levels (F(1,23) = 0.6509, *p* = 0.4280) and confirmed the absence of CB_2_ expression in samples from 5xFAD/CB_2_^-/-^ mice (Figure 3A; F(1,23) = 437.6, *p* < 0.0001). Second, we employed flow cytometry to quantify the binding of the selective fluorescent probe RO7246360 to the CB_2_ receptor; we found no changes induced by the chronic exposure to the agonist [F(1,22) = 0.02066, *p* = 0.8870] and confirmed the absence of CB_2_ protein in isolated microglia from 5xFAD/CB_2_^-/-^ mice (Figure 3B; F(1,22) = 55.62, *p* < 0.0001).

**FIGURE 4:**
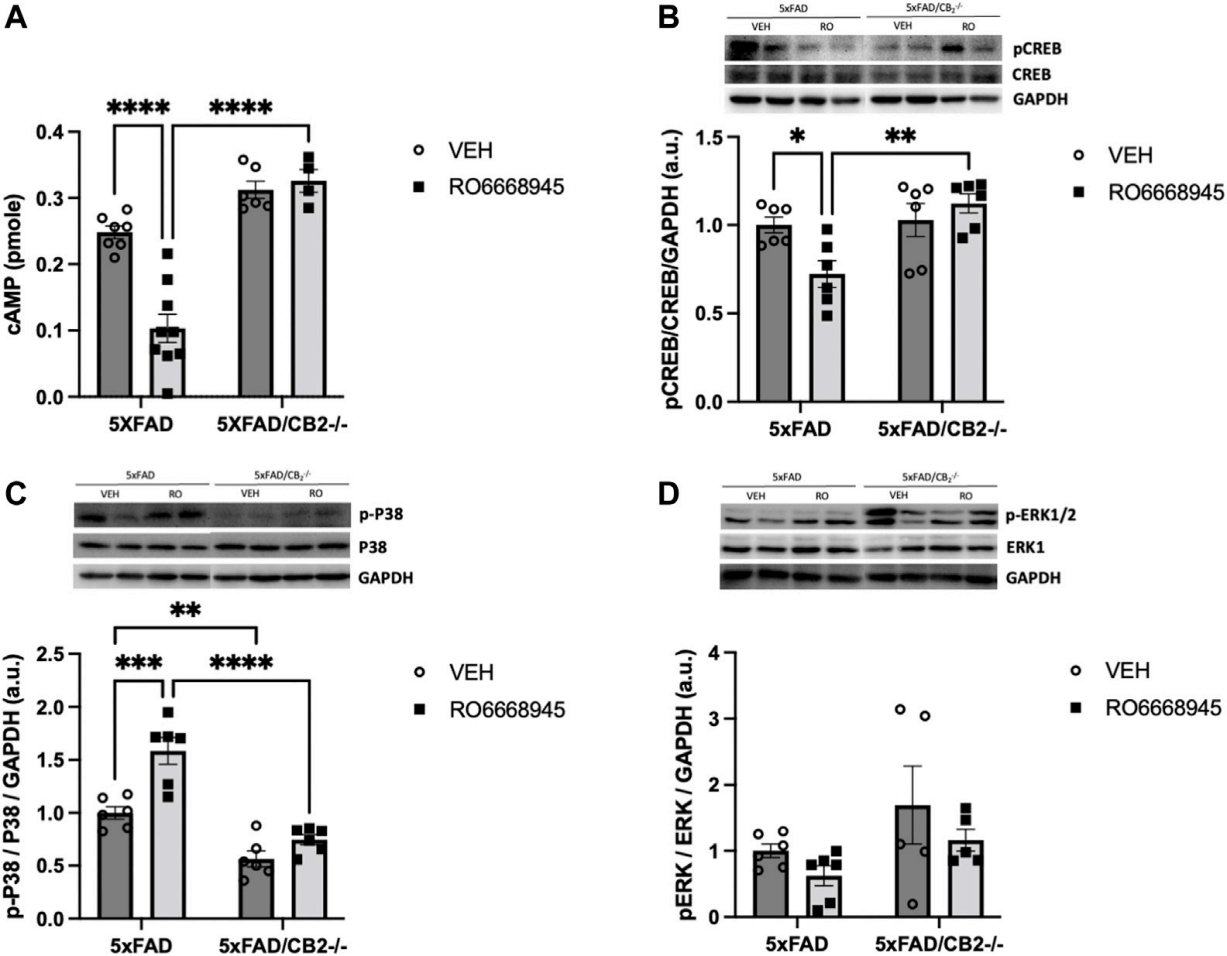
Signaling cascades regulated by the activation and deletion of cannabinoid CB_2_ receptors. **(A)** cAMP and p-CREB **(B)** levels were significantly decreased by the treatment with the CB_2_ agonist, remaining unaltered in samples from CB_2_-null mice. **(C)** p-p38MAPK levels were significantly elevated by the exposure to the agonist; in addition, samples from 5xFAD/CB_2_^-/-^ mice exhibited significantly lower levels. **(D)** p-ERK levels were not modified by the treatment with the CB_2_ agonist or the genetic deletion of the receptor. Two-way ANOVA followed by Tukey’s post-hoc test. *<*p*0.05; **<*p*0.01; ***<*p*0.001; ****<*p*0.0001. N = 4–9 mice per group. Data represent mean ± SEM.

We next analyzed the signaling cascades affected by CB_2_ activation or deletion (Figure 4). We found that the CB_2_ agonist had a significant impact on cAMP levels [Figure 4A; F(1,19) = 8.851, *p* = 0.0078]. Post-hoc analysis revealed a decrease in cAMP in 5xFAD/CB_2_^EGFP/f/f^ mice as a consequence of the treatment (*p* < 0.0001) that was absent in 5xFAD/CB_2_^-/-^ mice (*p* = 0.9802). No differences due to the genotype were observed in vehicle-treated mice (*p* = 0.2537), although were significant between RO6866945-treated 5xFAD/CB_2_^EGFP/f/f^ vs. 5xFAD/CB_2_^-/-^ mice (*p* < 0.0001).

Regarding pCREB levels (Figure 4B), genotype had a significant effect [F(1,20) = 9.370, *p* = 0.0062]. Post-hoc analysis revealed that the agonist significantly decreased pCREB levels in 5xFAD/CB_2_^EGFP/f/f^ mice (*p* = 0.0492) but not in 5xFAD/CB_2_^-/-^ mice (*p* = 0.7765). No differences due to the genotype were observed in vehicle-treated mice (*p* = 0.9917), although were significant between RO6866945-treated 5xFAD/CB_2_^EGFP/f/f^ *vs*. 5xFAD/CB_2_^−/−^ mice (*p* = 0.0033).

p-p38MAPK levels (Figure 4C) were modified by the treatment with RO6866945 [F(1,20) = 21.77, *p* = 0.0001], by genotype [F(1,20) = 60.28, *p* < 0.0001] and by the interaction of both factors [F(1,20) = 6.088, *p* = 0.0228]. p-p38MAPK was increased in 5xFAD/CB_2_^EGFP/f/f^ mice as a consequence of CB_2_ activation by the agonist (*p* = 0.0003) and exhibited significantly lower levels in samples from both vehicle- and RO6866945-treated CB_2_-lacking mice (*p* = 0.0064 and *p* < 0.0001, respectively). These observations highlight the selectivity of RO6866945 as a CB_2_-selective agonist and suggest a putative constitutive activation of p-38MAPK signaling cascade by CB_2_ receptors in the context of AD.

Finally, p-ERK levels remained unaltered after treatment with the agonist [F(1,18) = 2.377, *p* = 0.1405] as well as in 5xFAD/CB_2_^−/−^ mice (Figure 4D; F(1,18) = 4.339, *p* = 0.0518).

### CB_2_-Lacking Mice Express Lower Levels of Iba1 and Exhibit Impaired Phagocytic Activity

**FIGURE 5:**
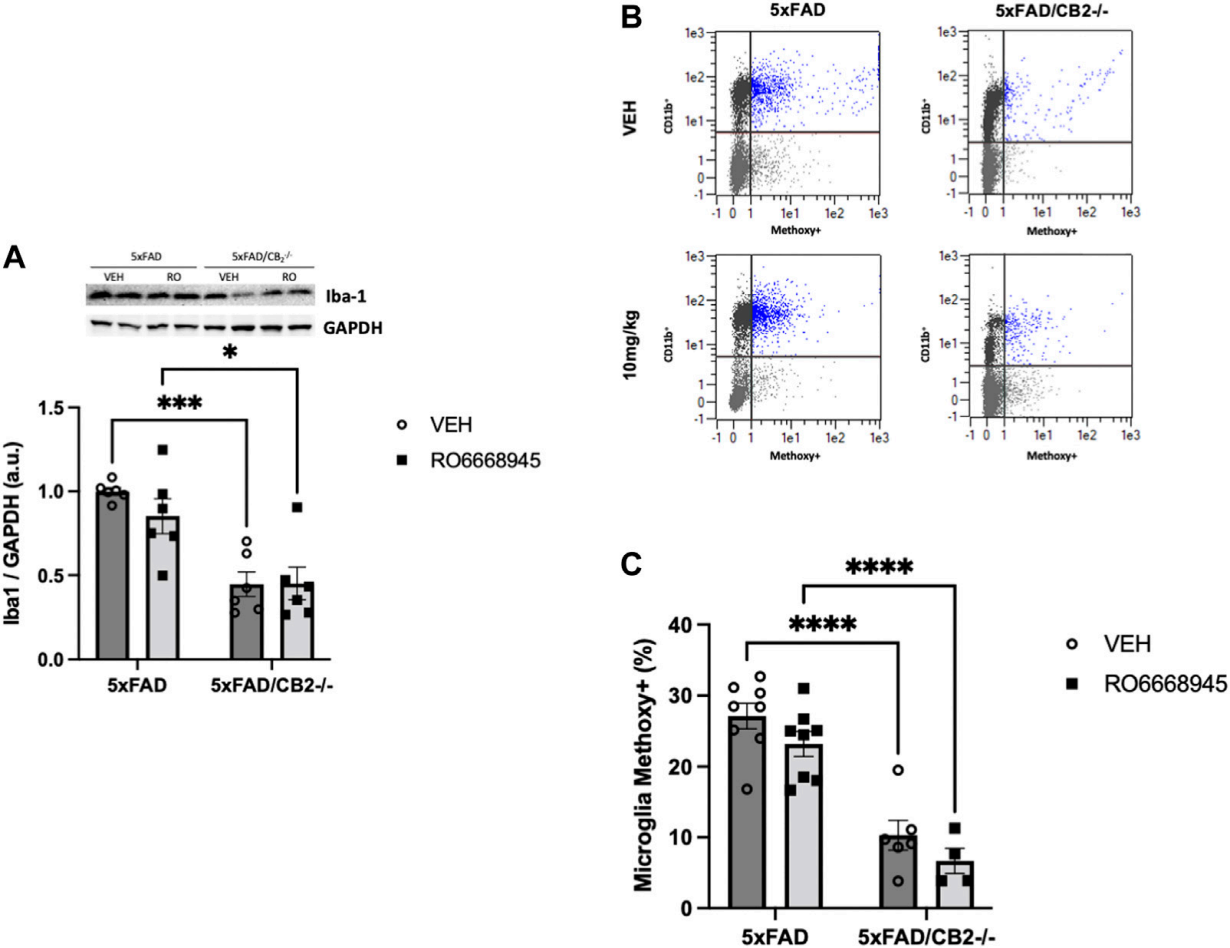
Iba1+ microglia and phagocytic activity is decreased after genetic deletion of the cannabinoid CB_2_ receptor. Analysis of cortices by western blot **(A)** and flow cytometry **(B,C)** revealed no changes associated to the treatment with the agonist together with a significant decrease in Iba1+ microglia **(A)**. Phagocytic activity **(B,C)** of microglia was significantly impaired in 5xFAD/CB_2_^-/-^ mice. Scatter plots of CD11b isolated microglia after intraperitoneal administration of methoxy-X04 are shown **(B)**. Phagocytic capacity was calculated as percentage of methoxy-X04+/CD11b+/CD45lo cells to CD11b+/CD45lo cells **(C)**. Two-way ANOVA followed by Tukey’s post-hoc test. *<*p*0.05. **<*p*0.01; ***<*p*0.001; ****<*p*0.0001. N = 5–6 mice per group. Data represent mean ± SEM.

As microglia are the main source of cannabinoid CB_2_ receptors in the brain of 5xFAD/CB_2_^EGFP/f/f^ mice, we analyzed the putative changes triggered in these cells by the activation of the receptor and by its genetic deletion (Figure 5). We found no changes in Iba1+ microglia (Figure 5A; F(1,20) = 0,7931, *p* = 0.3837) nor in its phagocytic activity (measured by its ability to internalize methoxy-X04-stained amyloid; Figures 5B,C; F(1,22) = 3.602, *p* = 0.0709) derived from CB_2_ activation by the agonist. However, significant differences were evident between 5xFAD/CB_2_^EGFP/f/f^ and 5xFAD/CB_2_^-/-^ microglia; thus, we found a decrease in Iba1+ microglia abundance [Figure 5A; F(1,20) = 34.95, *p* < 0.0001] as well as an impairment in its phagocytic activity (Figure 5B; F(1,22) = 69.96, *p* < 0.0001).

### The Activation as Well as the Genetic Deletion of CB_2_ Receptors Modify Amyloid Metabolism *in vivo*

**FIGURE 6:**
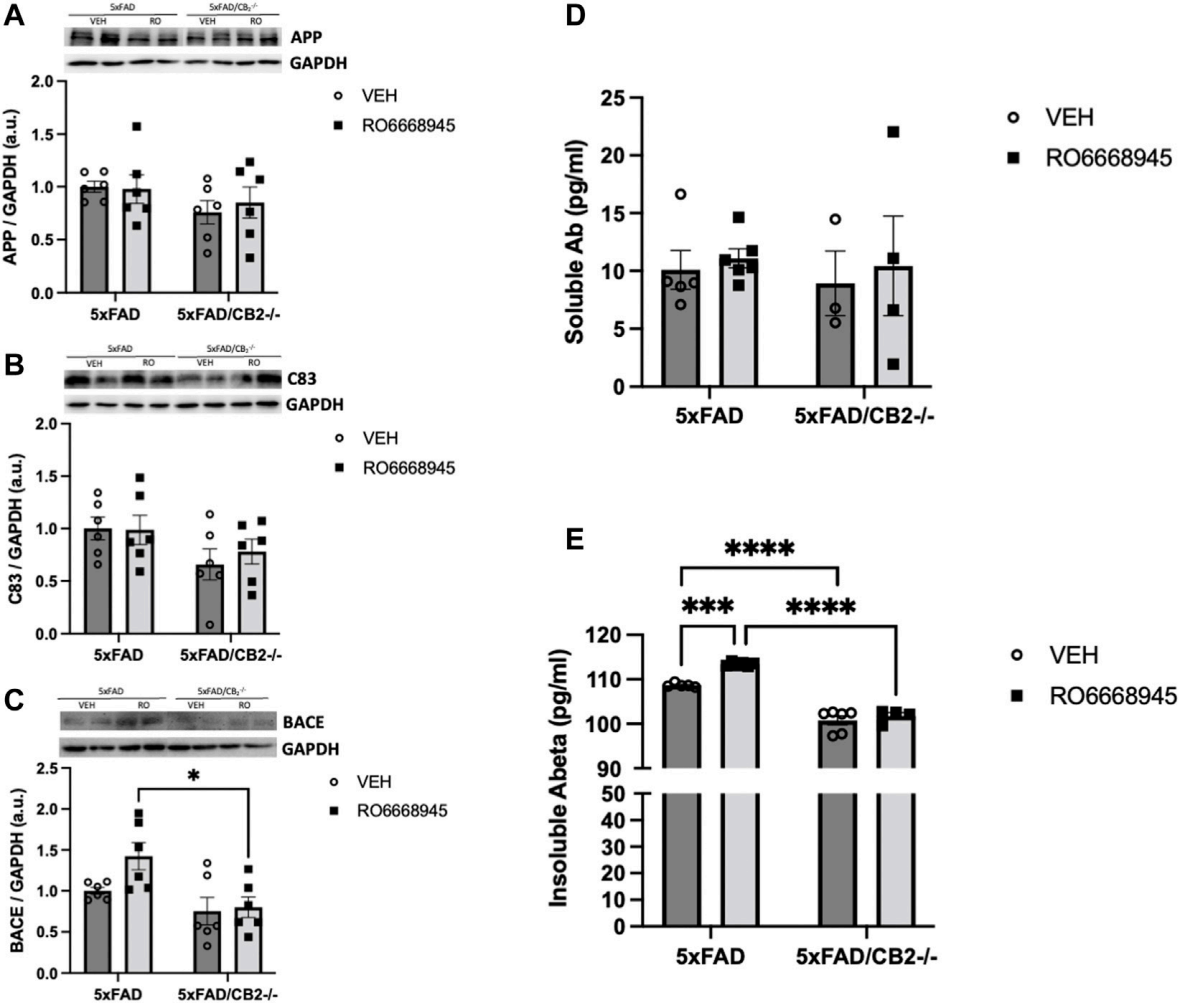
Cannabinoid CB_2_ receptors modulate amyloid dynamics *in vivo*. No changes were evident after treatment in APP **(A)**, C83 **(B)** or BACE1 **(C)**, as measured by western blot. Soluble amyloid levels **(D)** remained also unaltered, while those of insoluble amyloid **(E)** were significantly increased by the exposure to the CB_2_ agonist and decreased in 5xFAD/CB_2_^-/-^ mice. Two-way ANOVA followed by Tukey’s post-hoc test. *<*p*0.05; ****<*p*0.0001. N = 4-6 mice per group. Data represent mean ± SEM.

We next measured the impact of CB_2_ modulation on Aβ levels. To that end, we quantified several amyloid-related peptides (APP, C83 and BACE1) as well as the soluble and insoluble forms of Aβ_1-42_, the main component of neuritic plaques (Figure 6). Our data showed no changes in APP [F(1,20) = 0.08911, *p* = 0.7684], C83 [F(1,20) = 0.1794, *p* = 0.6764] or BACE1 [F(1,20) = 3.026, *p* = 0.0973] after treatment with RO6866945. CB_2_ deletion induced significant differences in protein levels of BACE1 [F(1,20) = 10.34, *p* = 0.0043], but not in C83 (F(1,20) = 1.705, *p* = 0.2065) and APP [F(1,20) = 2.468, *p* = 0.1319].

Levels of soluble amyloid were unaltered after treatment with the CB_2_ agonist [F(1,14) = 0.2681, *p* = 0.6127] or genetic inactivation of the receptor [F(1,14) = 0.1408, *p* = 0.1731; Figure 6D]. Cortical amounts of insoluble amyloid, however, were significantly modified by both (Figure 6E). Thus, the treatment with RO6866945 led to a significant increase in insoluble amyloid levels [F(1,17) = 17.88, *p* = 0.0006] in 5xFAD/CB_2_^EGFP/f/f^ mice, while 5xFAD/CB_2_^−/−^ mice exhibited decreased levels of this peptide [F(1,17) = 209.3, *p* < 0.0001].

## Discussion

In the present manuscript we report a significant role of cannabinoid CB_2_ receptors in microglial functions and in the metabolism of Aβ in an animal model of Alzheimer’s disease (5xFAD). Specifically, we found that the absence of CB_2_ receptors decrease the total number of microglial cells as well as their ability to phagocytose Aβ and have a modulatory role in the accumulation of the insoluble form of this pathogenic peptide. Furthermore, our data suggest that microglial CB_2_ receptors may be constitutively activated in the context of AD, as indicated by p38 phosphorylation state.

Our present data confirm the increased expression of cannabinoid CB_2_ receptors in plaque-associated microglia (Benito et al., 2007a). By using electronic microscopy, we have observed that the presence of EGFP (expressed under the control of the *Cnr2* promoter region) was enhanced specifically in these cells, in 5xFAD/CB_2_^EGFP/f/f^ mice, while in controls remained low or undetectable in microglial cells as well as in other cell types (such as neurons or astrocytes). These observations match with previous data obtained from our group (Benito et al., 2007b; López et al., 2018) and from others (Savonenko et al., 2015; Spangenberg et al., 2019) and confirm the selective expression of CB_2_ receptors in activated microglial cells in the context of the chronic neuroinflammation triggered by amyloid accumulation.

These findings allow us to assume that the changes in signal transduction cascades observed after treatment with the CB_2_ agonist, RO6866945, or after the genetic deletion of the receptor are mostly derived from the modulation of microglial cells, although the contribution of other receptor’s populations located in different types of cells cannot be completely ruled out. It might be possible that, even when expressed at very low levels, CB_2_ receptors could modulate the activity of neurons and/or astrocytes, as has been reported by other authors (Onaivi et al., 2008; Espejo-Porras et al., 2019). In our hands, however, microglial CB_2_ receptors must play a major role in the observed changes in cAMP, CREB and p38MAPK signaling cascades. Interestingly, p38MAPK regulation might be under the tonic influence of CB_2_ receptors, as its activity was significantly reduced in CB_2_-lacking mice.

These data match with those recently reported by Reusch et al. (Reusch et al., 2022) regarding microglial phagocytosis and signaling cascade (p38MAPK) profiles. By using cultures of BV-2 and primary microglia cells, these authors found that CB_2_ receptors are necessary for TLR-mediated activation, as shown by gene transcription, morphological and functional (LPS/IFN-γ, CpG and Polyl:C stimulation) analysis and that p38MPK signaling was directly involved in the CB_2_-mediated regulation of TLR function. Thus, primary neonatal microglia from CB_2_^-/-^ exhibited a dysregulation of this intracellular route at the transcriptional level that was especially evident after challenge with LPS/IFN-γ and Polyl:C, with a significant reduction in the phosphorylation level of p38. Furthermore, the significant decrease in the phagocytic activity of CB_2_-lacking microglia we herein report may be also associated to the loss of TLR function, as these receptors are well-known for their critical role in the uptake and clearance of amyloid by these cells (Tahara et al., 2006). Finally, our present observations match well with our previously published study in which a decrease in methoxy-X04+ plaques in 5xFAD/CB_2_^−/−^ mice was found (López et al., 2018).

As a limitation of the present studies, only male mice were employed. The question on putative sex differences in the 5xFAD model has been recently addressed by Forner et al. (Forner et al., 2021). These authors performed a comprehensive analysis of pathology-associated changes in male and female 5xFAD mice and found that female mice develop the disease at an earlier age, exhibit more significant weight loss, higher levels of insoluble Aβ and improved motor performance in the rotarod test than their males counterparts. A trend to increased microgliosis was also observed.

The current view on the pathogenesis of AD indicates that the accumulation of Aβ is one of the main hallmarks of this disease, together with the formation of tau-enriched neurofibrillary tangles (Querfurth and LaFerla, 2010). Both factors contribute to a significant loss of active synaptic connections in the cortex and hippocampus, triggering the well-known symptoms of this disease, such as memory loss, cognitive decline, etc. The animal model that we have employed in the present studies exhibits an enhanced amyloidogenic status, leading to the production of increased amounts of Aβ at early stages of the mouse’s lifespan and to the formation of neuritic plaques as early as 3 months of age (Oakley et al., 2006).

Though still controversial, the role of microglia in the formation and accumulation of amyloid-enriched neuritic plaques seems very relevant (Song and Colonna, 2018). It is thought that, in the context of AD and as the presence of increased species of Aβ extends in time, activated microglia become a relevant contributor to neuronal damage mainly by secreting elevated amounts of cytokines, ROS, and other mediators, and by losing their ability to phagocytose and degrade these pathological peptides (Song and Colonna, 2018). Importantly, microglia are thought to perform a “shielding” task by effectively surrounding neuritic plaques and thus preventing the expansion of the damage in the brain parenchyma (Condello et al., 2015). The complex role of microglia has been recently highlighted by recent reports showing that *in vivo* depletion of microglia (for instance, by the administration of antagonists of colony stimulating factor receptor-1, CSFR1, to mice) significantly alters plaque dynamics in the mouse brain. Spangenberg et al. (2019) and, very recently, Casali et al. (2020) have shown that microglia depletion prevents the formation of Aβ-enriched neuritic plaques and that microglia restoration favors its compact structure (Spangenberg et al., 2019; Casali et al., 2020). Our observations that CB_2_-deficient AD mice exhibit a decreased phagocytic activity combined with a decrease in cortical insoluble amyloid levels are suggestive of a role of CB_2_ receptors in plaque dynamics, in which their activation could contribute to a compaction of amyloid plaques while their deletion could lead to a more diffuse appearance. A similar effect has been described in TREM2-lacking mice, suggesting an impairment in microglial function (Wang et al., 2016).

Finally, it is important to note that other authors have reported conflicting data associated to the genetic deletion of cannabinoid CB_2_ receptors, different to those reported here. Koppel et al. (2014) used J20 APP mice to study the effects of CB_2_ genetic inactivation and found increased levels of soluble amyloid and plaques as well as enhanced plaque-associated microgliosis. In line with these data, Aso et al. (2016) also found significant increases in Aβ1-40 as well as in amyloid deposition in the APP/PS1 mouse model of AD. Wu et al. (2017) reported a stimulatory effect of a CB_2_ agonist on amyloid clearance combined with decreased microgliosis in the hippocampus of APP/PS1 mice. Conversely, Schmöle et al. (2015); Schmöle et al. (2018) found decreased microgliosis and amyloid levels as a consequence of CB_2_ deletion in APP/PS1 mice. Most of these studies also revealed no CB_2_-mediated effects on spatial memory. This variability regarding the effects of cannabinoid CB_2_ receptors may be partially explained by the variety of AD mouse models employed in these studies but may be also suggestive of the subtle and limited effects of modulating the activity of these receptors, as well as may reflect putative adaptive responses in constitutive knock-out models.

## Conclusion

We have confirmed (by immunoelectron microscopy) microglia as the main source of cannabinoid CB_2_ receptors in the 5xFAD/CB_2_^EGFP/f/f^ mouse model of AD. In addition, we have found that these receptors regulate the ability of these cells to phagocytose amyloid peptides *in vivo* and, probably in direct relation with this, in the composition of amyloid species in the brain. These data thus suggest a role for microglial cannabinoid CB_2_ receptors in the initiation, maintenance and removal of plaques and open new venues for the microglia-based therapeutic approaches in AD.

## Data Availability

The original contributions presented in the study are included in the article/supplementary material, further inquiries can be directed to the corresponding author.

## Abbreviations

Aβ, Amyloid beta; AD, Alzheimer’s Disease; ALS, Amyotrophic Lateral Sclerosis; APP, Amyloid Precursor Protein; BACE1, Beta-site Amyloid Precursor Protein Cleaving Enzyme 1; cAMP, Cyclic adenosine monophosphate; CNS, Central Nervous System; CREB, cAMP Response Element-Binding; CSFR1, Colony Stimulating Factor Receptor-1; EGFP, Enhanced Green Fluorescent Protein; ERK1/2, Extracellular Signal-Regulated Kinases ½; GABA-A, Gamma-Amino Butyric Acid Receptor Subunit Alpha-1; GAPDH, Glyceraldehyde-3-Phosphate Dehydrogenase; GPCRs, G-protein coupled receptors; HRP, Horseradish Peroxidase; Iba1, Ionized Calcium Binding Adapter Molecule 1; MAPK, Mitogen-Activated Protein Kinases; TREM2, Triggering Receptor Expressed On Myeloid Cells 2.

## References

[B1] Aso E. Andrés-Benito P. Carmona M. Maldonado R. Ferrer I. (2016). Cannabinoid Receptor 2 Participates in Amyloid-β Processing in a Mouse Model of Alzheimer's Disease but Plays a Minor Role in the Therapeutic Properties of a Cannabis-Based Medicine. J. Alzheimers Dis. 51, 489–500. 10.3233/JAD-150913 26890764

[B2] Benito C. Núñez E. Pazos M. R. Tolón R. M. Romero J. (2007a). The Endocannabinoid System and Alzheimer's Disease. Mol. Neurobiol. 36, 75–81. 10.1007/s12035-007-8006-8 17952652

[B3] Benito C. Núñez E. Tolón R. M. Carrier E. J. Rábano A. Hillard C. J. (2003). Cannabinoid CB2 Receptors and Fatty Acid Amide Hydrolase Are Selectively Overexpressed in Neuritic Plaque-Associated Glia in Alzheimer's Disease Brains. J. Neurosci. 23, 11136–11141. 10.1523/jneurosci.23-35-11136.2003 14657172 PMC6741043

[B4] Benito C. Romero J. P. Tolón R. M. Clemente D. Docagne F. Hillard C. J. (2007b). Cannabinoid CB1 and CB2 Receptors and Fatty Acid Amide Hydrolase are Specific Markers of Plaque Cell Subtypes in Human Multiple Sclerosis. J. Neurosci. 27, 2396–2402. 10.1523/JNEUROSCI.4814-06.2007 17329437 PMC6673484

[B5] Cabañero D. Ramírez-López A. Drews E. Schmöle A. Otte D. M. Wawrzczak-Bargiela A. (2020). Protective Role of Neuronal and Lymphoid Cannabinoid CB2 Receptors in Neuropathic Pain. Elife 9, e55582. 10.7554/eLife.55582 32687056 PMC7384863

[B6] Casali B. T. MacPherson K. P. Reed-Geaghan E. G. Landreth G. E. (2020). Microglia Depletion Rapidly and Reversibly Alters Amyloid Pathology by Modification of Plaque Compaction and Morphologies. Neurobiol. Dis. 142, 104956. 10.1016/j.nbd.2020.104956 32479996 PMC7526856

[B7] Condello C. Yuan P. Schain A. Grutzendler J. (2015). Microglia Constitute a Barrier that Prevents Neurotoxic Protofibrillar Aβ42 Hotspots Around Plaques. Nat. Commun. 6, 6176. 10.1038/ncomms7176 25630253 PMC4311408

[B8] Deczkowska A. Keren-Shaul H. Weiner A. Colonna M. Schwartz M. Amit I. (2018). Disease-Associated Microglia: A Universal Immune Sensor of Neurodegeneration. Cell 173, 1073–1081. 10.1016/j.cell.2018.05.003 29775591

[B9] Espejo-Porras F. García-Toscano L. Rodríguez-Cueto C. Santos-García I. de Lago E. Fernandez-Ruiz J. (2019). Targeting Glial Cannabinoid CB2 Receptors to Delay the Progression of the Pathological Phenotype in TDP-43 (A315T) Transgenic Mice, a Model of Amyotrophic Lateral Sclerosis. Br. J. Pharmacol. 176, 1585–1600. 10.1111/bph.14216 29574689 PMC6487601

[B10] Forner S. Kawauchi S. Balderrama-Gutierrez G. Kramár E. A. Matheos D. P. Phan J. (2021). Systematic Phenotyping and Characterization of the 5xFAD Mouse Model of Alzheimer's Disease. Sci. Data 8, 270. 10.1038/s41597-021-01054-y 34654824 PMC8519958

[B11] Galán-Ganga M. Rodríguez-Cueto C. Merchán-Rubira J. Hernández F. Ávila J. Posada-Ayala M. (2021). Cannabinoid Receptor CB2 Ablation Protects against TAU Induced Neurodegeneration. Acta Neuropathol. Commun. 9, 90. 10.1186/s40478-021-01196-5 34001284 PMC8130522

[B12] Hampel H. Hardy J. Blennow K. Chen C. Perry G. Kim S. H. (2021). The Amyloid-β Pathway in Alzheimer's Disease. Mol. Psychiatry 26, 5481–5503. 10.1038/s41380-021-01249-0 34456336 PMC8758495

[B13] He X. H. Galaj E. Bi G. H. He Y. Hempel B. Wang Y. L. (2021). β-Caryophyllene, an FDA-Approved Food Additive, Inhibits Methamphetamine-Taking and Methamphetamine-Seeking Behaviors Possibly via CB2 and Non-CB2 Receptor Mechanisms. Front. Pharmacol. 12, 722476. 10.3389/fphar.2021.722476 34566647 PMC8458938

[B14] Komorowska-Müller J. A. Schmöle A.-C. (2020). CB2 Receptor in Microglia: The Guardian of Self-Control. Int. J. Mol. Sci. 22, 19. 10.3390/ijms22010019 PMC7792761 33375006

[B15] Koppel J. Vingtdeux V. Marambaud P. d'Abramo C. Jimenez H. Stauber M. (2014). CB2 Receptor Deficiency Increases Amyloid Pathology and Alters Tau Processing in a Transgenic Mouse Model of Alzheimer's Disease. Mol. Med. 20, 29–36. 10.2119/molmed.2013.00140.revised 24722782 PMC3951462

[B16] López A. Aparicio N. Pazos M. R. Grande M. T. Barreda-Manso M. A. Benito-Cuesta I. (2018). Cannabinoid CB2 Receptors in the Mouse Brain: Relevance for Alzheimer's Disease. J. Neuroinflammation 15, 158. 10.1186/s12974-018-1174-9 29793509 PMC5968596

[B17] Maresz K. Carrier E. J. Ponomarev E. D. Hillard C. J. Dittel B. N. (2005). Modulation of the Cannabinoid CB2 Receptor in Microglial Cells in Response to Inflammatory Stimuli. J. Neurochem. 95, 437–445. 10.1111/j.1471-4159.2005.03380.x 16086683

[B18] Mecha M. Carrillo-Salinas F. J. Feliú A. Mestre L. Guaza C. (2016). Microglia Activation States and Cannabinoid System: Therapeutic Implications. Pharmacol. Ther. 166, 40–55. 10.1016/j.pharmthera.2016.06.011 27373505

[B19] Mechoulam R. Parker L. A. (2013). The Endocannabinoid System and the Brain. Annu. Rev. Psychol. 64, 21–47. 10.1146/annurev-psych-113011-143739 22804774

[B20] Nimmerjahn A. Kirchhoff F. Helmchen F. (2005). Resting Microglial Cells Are Highly Dynamic Surveillants of Brain Parenchyma *In Vivo* . Science 308, 1314–1318. 10.1126/science.1110647 15831717

[B21] Oakley H. Cole S. L. Logan S. Maus E. Shao P. Craft J. (2006). Intraneuronal Beta-Amyloid Aggregates, Neurodegeneration, and Neuron Loss in Transgenic Mice with Five Familial Alzheimer's Disease Mutations: Potential Factors in Amyloid Plaque Formation. J. Neurosci. 26, 10129–10140. 10.1523/JNEUROSCI.1202-06.2006 17021169 PMC6674618

[B22] Onaivi E. S. Ishiguro H. Gong J. P. Patel S. Meozzi P. A. Myers L. (2008). Functional Expression of Brain Neuronal CB2 Cannabinoid Receptors are Involved in the Effects of Drugs of Abuse and in Depression. Ann. N. Y. Acad. Sci. 1139, 434–449. 10.1196/annals.1432.036 18991891 PMC3922202

[B23] Ouali Alami N. Schurr C. Olde Heuvel F. Tang L. Li Q. Tasdogan A. (2018). NF-κB Activation in Astrocytes Drives a Stage-Specific Beneficial Neuroimmunological Response in ALS. EMBO J. 37 (16), e98697. 10.15252/embj.201798697 29875132 PMC6092622

[B24] Pertwee R. G. Howlett A. C. Abood M. E. Alexander S. P. Di Marzo V. Elphick M. R. (2010). International Union of Basic and Clinical Pharmacology. LXXIX. Cannabinoid Receptors and Their Ligands: beyond CB₁ and CB₂. Pharmacol. Rev. 62, 588–631. 10.1124/pr.110.003004 21079038 PMC2993256

[B25] Puente N. Río I. B. Achicallende S. Nahirney P. C. Grandes P. (2019). High-Resolution Immunoelectron Microscopy Techniques for Revealing Distinct Subcellular Type 1 Cannabinoid Receptor Domains in Brain. Bio Protoc. 9, e3145. 10.21769/BioProtoc.3145 PMC7854156 33654890

[B26] Querfurth H. W. LaFerla F. M. (2010). Alzheimer's Disease. N. Engl. J. Med. 362, 329–344. 10.1056/NEJMra0909142 20107219

[B27] Reusch N. Ravichandran K. A. Olabiyi B. F. Komorowska-Müller J. A. Hansen J. N. Ulas T. (2022). Cannabinoid Receptor 2 Is Necessary to Induce Toll-like Receptor-Mediated Microglial Activation. Glia 70, 71–88. 10.1002/glia.24089 34499767

[B28] Rodríguez-Cueto C. Gómez-Almería M. García Toscano L. Romero J. Hillard C. J. Lago E. (2021). Inactivation of the CB 2 Receptor Accelerated the Neuropathological Deterioration in TDP‐43 Transgenic Mice, a Model of Amyotrophic Lateral Sclerosis. Brain Pathol. 31, e12972. 10.1111/bpa.12972 33983653 PMC8549023

[B29] Sarott R. C. Westphal M. V. Pfaff P. Korn C. Sykes D. A. Gazzi T. (2020). Development of High-Specificity Fluorescent Probes to Enable Cannabinoid Type 2 Receptor Studies in Living Cells. J. Am. Chem. Soc. 142, 16953–16964. 10.1021/jacs.0c05587 32902974

[B30] Savonenko A. V. Melnikova T. Wang Y. Ravert H. Gao Y. Koppel J. (2015). Cannabinoid CB2 Receptors in a Mouse Model of Aβ Amyloidosis: Immunohistochemical Analysis and Suitability as a PET Biomarker of Neuroinflammation. PLoS One 10, e0129618. 10.1371/journal.pone.0129618 26086915 PMC4472959

[B31] Schmöle A. C. Lundt R. Ternes S. Albayram Ö. Ulas T. Schultze J. L. (2015). Cannabinoid Receptor 2 Deficiency Results in Reduced Neuroinflammation in an Alzheimer's Disease Mouse Model. Neurobiol. Aging 36, 710–719. 10.1016/j.neurobiolaging.2014.09.019 25443294

[B32] Schmöle A. C. Lundt R. Toporowski G. Hansen J. N. Beins E. Halle A. (2018). Cannabinoid Receptor 2-Deficiency Ameliorates Disease Symptoms in a Mouse Model with Alzheimer's Disease-Like Pathology. J. Alzheimers. Dis. 64, 379–392. 10.3233/JAD-180230 29865078

[B33] Song W. M. Colonna M. (2018). The Identity and Function of Microglia in Neurodegeneration. Nat. Immunol. 19, 1048–1058. 10.1038/s41590-018-0212-1 30250185

[B34] Spangenberg E. Severson P. L. Hohsfield L. A. Crapser J. Zhang J. Burton E. A. (2019). Sustained Microglial Depletion with CSF1R Inhibitor Impairs Parenchymal Plaque Development in an Alzheimer's Disease Model. Nat. Commun. 10, 3758. 10.1038/s41467-019-11674-z 31434879 PMC6704256

[B35] Tahara K. Kim H. D. Jin J. J. Maxwell J. A. Li L. Fukuchi K. (2006). Role of Toll-Like Receptor Signalling in Abeta Uptake and Clearance. Brain 129, 3006–3019. 10.1093/brain/awl249 16984903 PMC2445613

[B36] Wang Y. Ulland T. K. Ulrich J. D. Song W. Tzaferis J. A. Hole J. T. (2016). TREM2-Mediated Early Microglial Response Limits Diffusion and Toxicity of Amyloid Plaques. J. Exp. Med. 213, 667–675. 10.1084/jem.20151948 27091843 PMC4854736

[B37] Wu J. Hocevar M. Foss J. F. Bie B. Naguib M. (2017). Activation of CB2 Receptor System Restores Cognitive Capacity and Hippocampal Sox2 Expression in a Transgenic Mouse Model of Alzheimer's Disease. Eur. J. Pharmacol. 811, 12–20. 10.1016/j.ejphar.2017.05.044 28551012

[B38] Zhang H. Y. Gao M. Liu Q. R. Bi G. H. Li X. Yang H. J. (2014). Cannabinoid CB2 Receptors Modulate Midbrain Dopamine Neuronal Activity and Dopamine-Related Behavior in Mice. Proc. Natl. Acad. Sci. U. S. A. 111, E5007–E5015. 10.1073/pnas.1413210111 25368177 PMC4246322

